# Apatinib-Induced Hand–Foot Skin Reaction in Chinese Patients With Liver Cancer

**DOI:** 10.3389/fonc.2021.624369

**Published:** 2021-04-26

**Authors:** Hui Xia, Cheng Zhou, Zhaoxia Luo, Ping Zhang, Liping Zhu, Zhao Gong

**Affiliations:** ^1^ Department of Hepatobiliary Surgery, Wuhan No. 1 Hospital, Wuhan, China; ^2^ Department of Dermatology, Wuhan No. 1 Hospital, Wuhan, China

**Keywords:** apatinib, hand–foot skin reaction, mechanisms, management, Chinese****

## Abstract

Apatinib, an anti-tumor drug selectively targeting VEGFR2 (Vascular Endothelia Growth Factor Recpetor-2), has been proven effective in Chinese patients with liver cancer. Generally, treatment with apatinib achieves 16.1% of the overall objective remission rate (ORR) and 55.83% of the disease control rate (DCR) in Chinese patients with liver cancer. However, the prevalence of apatinib-induced hand–foot skin reaction (AI-HFSR) is noticeably high. The incidence of AI-HFSR is about 50.5%, of which Grades 1/2 and 3 are 38.8 and 11.6%, respectively. In addition, potential molecular mechanisms underlying the development of AI-HFSR are poorly understood and urgently needed to be investigated histologically. In this review, we summarize and review the current efficacy of apatinib and the prevalence of AI-HFSR in Chinese patients with liver cancer. Besides, we postulate the potential mechanisms underlying the development of AI-HFSR and discuss the optimal clinical management for this unwanted cutaneous side effect.

## Introduction

Apatinib is a tyrosine kinase inhibitor (TKI) independently developed in China with an IC50 of 1.0 nmol/L. It can efficiently and competitively bind to the intracellular ATP binding site of VEGFR-2 (Vascular Endothelial Growth Factor Recpetor-2) protein, thus blocking the downstream signal transduction as well as the migration and proliferation of vascular endothelial cells. As a result, the process of angiogenesis, which is necessary and crucial for tumor growth and progression, is inhibited. VEGFR-2 blockade constitutes the fundamental anti-tumor mechanisms of apatinib. In 2014, apatinib was approved by the Chinese Drug and Food Administration (CDFA) as a second line therapy for advanced gastric cancer or adenocarcinoma of the gastro-esophagus junction.

China has a large number of patients with advanced liver cancer (1). Sorafenib, the traditional and widely-used first-line targeted medication for this disease, is of unsatisfactory benefits and costs extremely high for Chinese patients. Recently, a number of clinical and basic research projects have been funded in China to investigate the potential therapeutic efficacy and clinical complications of apatinib for the treatment of liver cancer. Based on published literatures, however, the prevalence of apatinib-induced HFSR (AI-HFSR) could be as high as 81.8% (2). In some severe cases, AI-HFSR leads to inevitable withdrawal or interruption of the continuous medication, which may weaken the survival benefits of the patients (3). Meanwhile, HFSR shows up as a typical interdisciplinary disease, which usually confuses physicians when handling with it in clinical practice. For these reasons, we here reviewed the literatures for the progress achieved in recent years on various aspects of prevalence, characteristics, diagnosis, mechanisms and treatment of HFSR in Chinese patients with liver cancer.

## Prevalence of AI-HFSR in Chinese Patients With Liver Cancer

Although, apatinib has only been approved as the second line of therapy for gastric adenocarcinoma so far in China, its promising effects on the treatment of hepatocellular carcinoma (HCC) have been preliminarily shown in a Phase 2 clinical trial (4). Meanwhile, the performance of conventional chemotherapy for advanced liver cancer still remains unsatisfactory. All these made apatinib standing in the spotlight. Therefore, increasing number of clinical studies have been designed to assess its value in treating liver cancer, which ranks the sixth malignant cancer in the world (5), and the third in China (1). With the accumulation of clinical experiences, however, frequent appearance of AI-HFSR during the treatment with apatinib has aroused our intensive concern. In this review, we collected data about the prevalence and clinical outcomes of AI-HFSR in Chinese patients with liver cancer who have received the treatment of apatinib since 2014.

As shown in **Table 1**, the incidence of AI-HFSR in Chinese patients with liver cancer is 50.5% (249/493). After excluding the studies by Zhu et al. (7) and Han et al. (13) (due to the lack of information on AI-HFSR grading), incidence of Grades I/II was 38.8% (174/448), and 11.6% (52/448) for Grade III. Actually, the overall incidence rates are slightly higher than that was previously reported in a phase II clinical study (4).

**Table 1:** Prevalence of AI-HFSR and treatment outcomes of apatinib in chinese patients with liver cancer.

Author	Publish Date	Research Type	Stage	Case (n)	Combined Therapy	Dosage	Therapy Outcomes				HFSR Incidence (%)		
							ORR(%)	DCR(%)	TTP/PFS(m)	OS(m)	Total	Grade I/II	Grade III
Qin et al. (4)	2014	Prospective	Adv	71	no	850 mg	8.57	48.57	4.42	9.71	41.4	35.7	5.7
				50	no	750 mg	0	37.25	3.32	9.82	29.4	21.6	7.8
Kou et al. (6)	2017.1	Case Report	Adv	1	c-TACE + Chemo.	500 mg							√
Lu et al. (7)	2017.5	Prospective	Mid/Adv	20	c-TACE	500 mg	35	60	12.5	N/A	55	50	5
Kong et al. (8)	2017.5	Prospective	Adv	22	No	500 mg/250 mg	40.9	81.8	10.4	N/A	81.8	68.2	13.6
Yu et al. (9)	2018.1	Prospective	Mid/Adv	31	No	500 mg	32.26	80.65	4.8	N/A	100	58.1	41.9
Zhen et al. (10)	2018.3	Retrospective	Unresec/Recur	32	No	250 mg/435 mg/500 mg	16	60	5	13	25	22	3
Yang et al. (11)	2018.10	Retrospective	Adv	25	c-TACE	250 mg	36	56	4.5	16.5	68	60	8
Wang et al. (12)	2018.11	Retrospective	Mid/Adv	34	No	750 mg	17.6	38.2	4.79	7.18	29.41	26.47	2.94
Han et al. (13)	2018.12	Case Report	Adv	1	c-TACE + OP + sorafenib	250 mg				19	√		
Yang et al. (14)	2019.1	Case Report	Adv	1	No	500 mg			12.5	N/A		√	
Zhu et al. (15)	2019.3	Prospective	Adv	44	c-TACE	500 mg	63.64	95.4	16.5	N/A	50	N/A	N/A
Fan et al. (16)	2019.4	Retrospective	Adv.	85	c-TACE	500 mg	28.2	58.7	6.1	12	52.9	29.4	23.5
Zhang et al. (17)	2019.5	Case Report	Recur	1	Camrelizumab	250 mg						√	
Liu et al. (18)	2019.7	Prospective	Mid/Adv	32	Deb-TACE	500 mg	68.8	90.6	9.5	22	28.1	28.1	0
Zhang et al. (17)	2019.7	Retrospective	Adv	43	No	500 mg	25.6	67.4	3	8	74.4	69.8	4.7

Adv, advanced stage; Mid, middle stage; Recur, recurrent; Unresec, unresectable; c-TACE, conventional transarterial chemoembolization; Deb-TACE, drug-eluting bead transarterial chemoembolization; ORR, objective remission rate; DCR, Disease Control Rate; TTP, Time to Progress; PFS, Progress Free Time; OS, Overall Survival.

When used as monotherapy, the objective remission rate (ORR) of apatinib was 16.61% (47/283) and the disease control rate (DCR) was 55.83% (158/283). When combined with TACE, the ORR increases to 43.69% (90/206), and the DCR goes up to 71.3% (147/206). Of note, apatinib in combination with Deb-TACE yielded a better outcome, with ORR and DCR reaching 68.8 and 90.6%, respectively, and an overall survival of 22 months.

To better understand AI-HFSR, in our opinion, efforts need to be made to shed more light on two aspects: the detailed molecular mechanisms of apatinib in the anti-tumor treatment of liver cancer; the histological features of AI-HFSR lesions in patients receiving apatinib therapy.

### Anti-Tumor Mechanisms of Apatinib in Liver Cancer

Many studies have been carried out to explore the potential anti-tumor mechanisms of apatinib. We categorize here the published literatures obtained from the online database on the basis of three aspects (**Table 2**).

**Table 2:** Pathways underlying the anticarcinogenic effect of apatinib on liver cancer.

Author	Publish Date	Journal	Pathway	*Vitro*	*Vivo*
Peng et al. (19)	2016.3	Oncotarget	PI3K/AKT/mTOR	CCA: RBE, SSP25	RBE
Jiang et al. (20)	2016.8	Chin J Clin Pharmacol	P53, Caspase-3, Caspase-8	HCC: HepG2	
Wen et al. (21)	2018.1	Pathol Res Pract	STAT3, BAX/Bcl-2	HCC: SMCC-7721	
Zhang et al. (22)	2018.1	Oncol Lett	PI3K/AKT	HCC: SMCC-7721, Bax, Caspase-9, Bcl-2	
Li et al. (23)	2018.2	Biochim Biophys Acta Mol Basis Dis	PDGFR-α, IGF-IR	CCA: HCCC-9810; HCC: Huh-7, Li-7, BEL-7402, Hep3b, HepG2	Hep3b
Yang et al. (11)	2018.6	Cancer Med	AKT, ERK1/2, G2/M	HCC: SK-Hep-1, HepG2, Hep3B, Huh-7, PLC/PRF/5, SMMC-7721; EC: HUVEC	PLC/PRF/5,SK-Hep-1
Huang et al. (24)	2018.11	BMC Gastroenterol	RAF/MEK/ERK, PI3K/AKT	CCA: QBC939, TFK-1	
Gu et al. (25)	2019.6	J Cell Biochem	Pyruvate, Alanine, Aspartate, Glutamate	HCC: HepG2	
Feng et al. (26)	2019.8	Cancer Sci	PPARα, 3-HB	HCC: HepG2	A549
Liao et al. (27)	2019.11	J Exp Clin Cancer Res	PI3K/AKT	HCC: SMMC-7721, MHCC-97H, HCCLM3, Hep-3B	SMMC-7721

CCA, Cholangiocarcinoma; HCC, Hepatocellular Carcinoma.

### Anti-Tumor Mechanisms in Hepatocellular Carcinoma

As discussed above, apatinib shows competitive binding to VEGFR-2, thus inhibiting the angiogenesis of vascular endothelium (11). Consequently and logically, impaired angiogenesis leads to tumor suppression. Several studies have shown that apatinib can also functionally suppress the STAT3, ERK1/2, and PI3K/AKT pathways (11, 22–24, 28), downregulate the expression of pro-survival mediators of PDGFα, IGF-IR, and Bcl-2 (22, 23, 28), and upregulate the expression of pro-apoptotic genes of Bax, p53, and Caspases 3, 8, and 9 (20, 22). Through these mechanisms, apatinib exerts its anti-tumor activity through promoting the apoptosis of hepatic tumor cells. Furthermore, apatinib has also been demonstrated to be able to arrest the proliferating cancer cells at the G2/M cell cycle stage (11), and promote the vascular normalization of tumors, thus enhancing the sensitivity of radiotherapy for HCC (27).

### Anti-Tumor Mechanisms in Cholangiocarcinoma

Huang et al. (24) showed that apatinib could suppress migration and invasion of CCA cells through inhibiting the RFA/MEK/ERK and PI3K/AKT signaling pathways. The study by Peng et al. (19) also showed that apatinib could inhibit the anti-apoptosis pathway of PI3K/AKT/mTOR signaling to promote the cellular death of CCA cells *in vitro*.

### Metabolism Regulation of Apatinib in Liver Cancer

Recently, researchers found that apatinib could also regulate the metabolic functions of liver cancer cells. For instance, Gu et al. (25) reported that apatinib reduces the intake of pyruvate, and retards the secretion of lactate in HepG2 cells. According to Feng’s study (26), apatinib has been demonstrated to enhance the synthesis of 3-hydroxybutyric acid (3HB), a metabolites inhibiting tumor growth both *in vitro* and *in vivo*, through activation of PPARα and promotion of fatty acid utilization. These recent studies provided clues to potential mechanism of metabolism regulation underlying the antitumor effect of apatinib besides its anti-angiogenic effect.

## Histology of HFSR

To the best of our knowledge, there are no studies elucidating the histological characteristics of AI-HFSR lesions. Reports on TKI-induced HFSR are also relatively lacking. Because of this, we referred to the histological data related to other TKIs, such as sorafenib, sunitinib, regorafenib etc. Based on the criteria of classification for HFSR which was proposed by the NCI (National Cancer Institute), the severity of HFSR is graded into three levels. The features of TKI-induced HFSR include: 1. Increased epidermal proliferation (papillomatosis, acanthosis); 2. Keratinocyte necrosis (eosinophilic body, vacuolar degeneration, confluent keratinocyte necrosis, and intraepidermal cleavage); 3. Keratinocyte over-proliferation (parakeratosis, retention of pyknotic nuclei). These characteristics are usually different from the chemotherapeutic drugs-induced HFS (Hand–Foot Syndrome), which is mainly manifested by necrosis of epithelial cells and injury of eccrine sweat glands.

Although modifications have been made on CTCAE version 3.0 (29) to make it more accurately in line with clinical practice, the histological description of HFSR in version 4.03 (30) is still incomplete and lacking in details. In 2009, Yang et al. (31) performed histological examination on eight cases of sorafenib-induced HFSR. They found eosinophilic bodies emerged in 80% of the samples. They explained the observed pathological changes as a secondary consequence to keratinocyte necrosis. The typical manifestation of HFSR is apoptosis of keratinocytes and satellitosis of lymphocytes. Besides, emergence of cavernous lesions and lack of neutrophilic infiltration, which resembles the histological features of immune response-mediated skin lesions, was also observed in HFSR (32). Despite of diverse characteristics, previous reports consistently found that lack of granulocytes infiltration and increased accumulation of lymphocytes in the dermis of skin occurs frequently during the development of HFSR (31, 33). In 2019, however, Sirka et al. (34) reported a grade III case of HFSR displaying a different histological signature. The results of biopsy discovered plenty of bullae within the stratum corneum and exuberant infiltration of neutrophils and fibrin, which was different from other reports. This implies that, in severe cases, neutrophils might be recruited when concomitant bacterial infection occurs. All in all, future histopathological studies are still needed to shed a deep insight into the features of TKI-induced HFSR.

### Potential Mechanisms Underlying AI-HFSR

Although the mechanism of HFSR remains poorly understood, several recognitions can be conceivably acquired: First, like other TKIs, AI-HFSR usually occurs in a dose-dependent manner which indicates that the development of AI-HFSR is not because of allergic reaction (35). Second, it is known that, chemotherapeutic drugs can be excreted *via* the eccrine sweat glands, through which exerting its toxic injury to the skin during the development of HFS. However, similar as TKIs like sorafenib, which is lipophilic and has been demonstrated to be undetectable from the sweat glands (36), apatinib-induced HFSR probably maintains a different mechanism of pathogenesis from that of chemotherapeutic drugs-induced HFS. Third, Bevzcizumab, a recombinant humanized anti-IgG1 antibody against VEGF, does not give rise to HFSR when used as monotherapy for patients (37). This implies that inhibition of VEGF/VEGFR signaling is not the main cause of apatinib-induced cutaneous side effects. Therefore, mechanisms presiding over the AI-HFSR seem to be a group of pathogenic factors.

Under physiological conditions, body parts, such as the fingers, palms, and soles, bear continuous pressure or frequent impact. In other words, they suffer from so called “subclinical trauma” (38). Chronic and persistent trauma requires constant tissue repair. However, following treatment with TKIs, the regenerative ability is compromised. VEGFR and PDGFR are suppressed by the TKIs, resulting in peripheral vascular endothelium dysfunction (33). Therefore, the capacity of cutaneous self-healing is severely impeded (39, 40). Histological investigation on HFSR shows massive keratinocytic apoptosis, perivascular lymphocytic infiltration, interface dermatitis without neutrophils participation, basilar vacuolar degeneration, and dyskeratosis. This supports the possibility of direct cytotoxic injury (41). Thus, the prevalence hypothesis is that HFSR is caused by drug extravasation from impaired dermic capillaries due to subclinical trauma. However, experimental evidence showing high concentration of apatinib in the skin of palms or soles is still missing.

Histologically, the coexistence of over proliferation and extensive necrosis in keratinocytes is a prominent feature of AI-HFSR. This brings up two questions that need to be answered.

First, does TKI directly promote proliferation of keratinocytes? On the contrary, clinical practice demonstrated that topical application of sunitinib could efficiently alleviate the keratosis of skin lesions in patients with psoriasis. Mechanically, Ye et al. (42) showed that sunitinib inhibits keratinocyte proliferation by reducing the pSTAT3 activity. Under physiological conditions, skin areas that are frequently exposed to continuous stress or friction prefer to form callus through keratinocyte hyperproliferation. The formed callus defensively disperse the pressure on this area and protect the deeper tissue from mechanical injury (43). Therefore, for AI-HFSR, possible mechanisms can be conceived that high dose usage of apatinib exerts an inhibitory effect on VEGFR/PDGFR, thus hindering the self-repair of the cutaneous tissues. As a result, damages caused by subclinical trauma will persist for the existence of handicapped regeneration induced by apatinib. This will subsequently provoke the proliferation of keratinocytes, thereby giving rise to the development of AI-HFSR. Furthermore, since the speed of cell proliferation has been significantly accelerated, there is not enough time for the proliferating keratinocytes to fully differentiate and become mature. Consequently, part of the regenerated keratinocytes is incompletely differentiated, and thereafter parakeratosis or dyskeratosis occurs (43–45). All the characteristics mentioned above are actually consistent with the histologic manifestations of AI-HFSR.

The second question is, in which pathway the TKIs lead keratinocytes to apoptosis? The papers by Zeng et al. (46) and Li et al. (47) confirmed that blocking the ERK1/2 signaling pathway in HaCaT, a human keratinocyte cell line, significantly retarded the inflammatory reaction of these cells *in vitro*. Meanwhile, experiments with apatinib showed that, high dose administration of apatinib exactly exert an inhibitory effect on ERK1/2 signaling activation (48). As such, apoptosis of keratinocytes is probably not mediated through the activation of ERK1/2 pathway and their associated inflammatory injury. Preclinical pharmacokinetic study indicated that sorafenib has a longer half-time in the skin (72.8 h), when compared with in other organs (20–36 h) (49). Eric et al. (50) found that keratinocytes uptake sorafenib by membrane transporter OAT6 (Organic Anion Transporter 6). The intracellularly transported sorafenib then targets MAP3K7 (Mitogen-activated Protein Kinase 3K7) and causes keratinocyte apoptosis. Another study also showed that inactivation of TAK1 (MAP3K7) was able to increase the accumulation of ROS (Reactive Oxygen Species) partly through c-Jun signaling pathway, by which inducing keratinocyte apoptosis (51). However, whether or not the OAT6-mediated drug transportation or the TAK1-c-Jun-ROS axis-regulated apoptosis also play a role in regulating the keratinocyte injury of AI-HFSR is worth interrogating in future studies.

**Figure 1:**
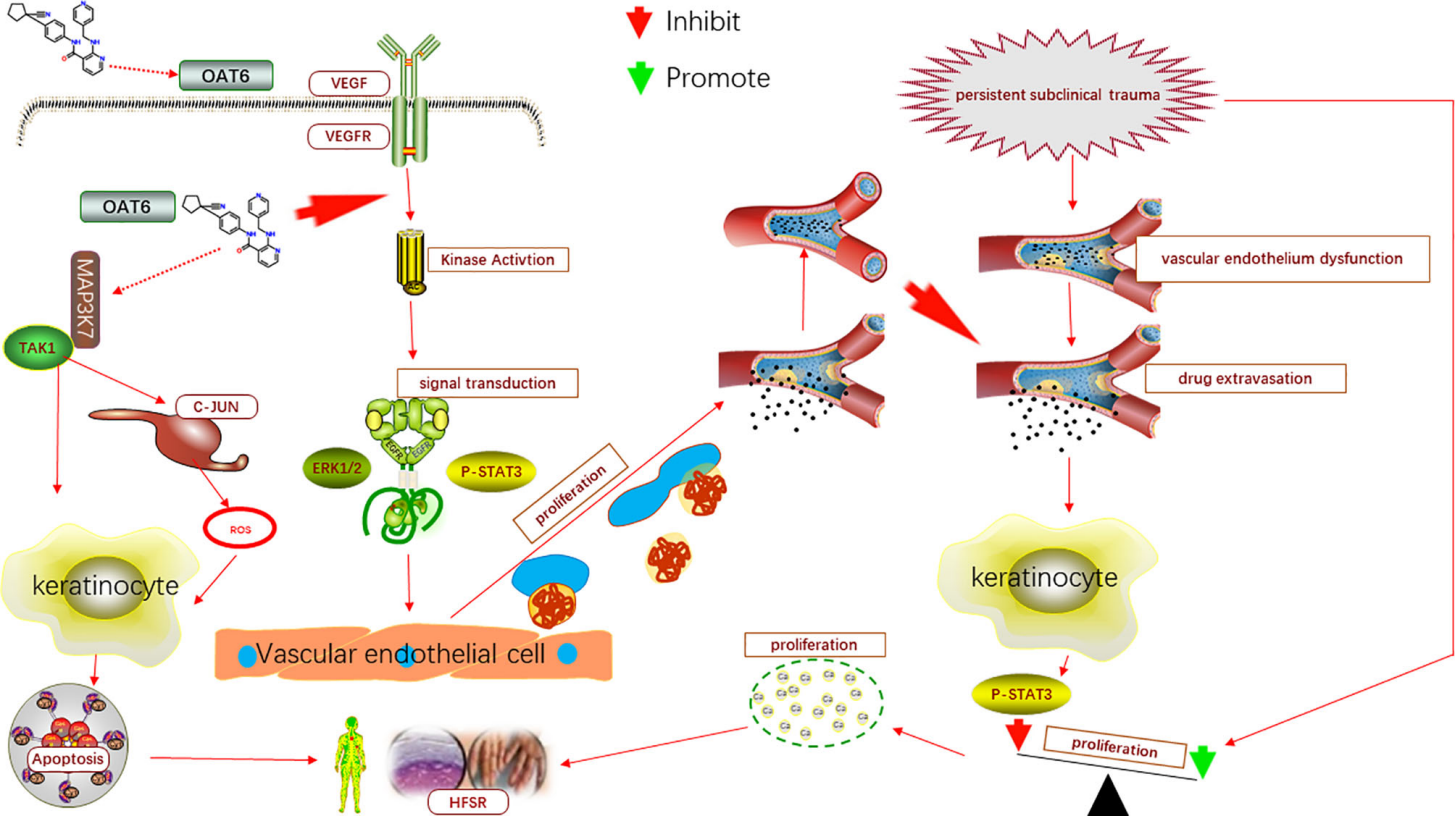
The model of AI-HFSR in liver cancer. By impeding the reparation of capillary, apatinib may cause the influence of subclinical damage persistence, which in turn, contribute to the over proliferation of keratinocytes; Apatinib might be uptaken by OAT6 on membrane of keratinocyte, and promote the apoptosis of keratinocyte per TAK1/C-JUN-ROS.

In conclusion, persistent existence of subclinical trauma, impaired vascular function and TKIs-induced keratinocyte apoptosis, are contributing factors collectively facilitating the development of AI-HFSR. The TKIs extravasated from damaged capillary vessels caused by persistent subclinical trauma impede the regeneration of endothelium per ERK1/2 or P-STAT3 pathway, and lead to the deterioration of capillary injury and extravasation. Although, TKIs has directly inhibitive effects on proliferation of keratinocyte by P-STAT3 signal path, but its’ strength is far more weaker than the opponent promoting effect derived from the persistent subclinical trauma, therefore, the keratinocytes present over proliferation in HFSR as the result. In addition, through the TAK1 and C-JUN/ROS pathway, TKIs could also promote the apoptosis of keratinocytes. Therefore, the injuries of capillary, over proliferation and apoptosis of keratinocytes compose the characteristic histology manifestations, which are very informative for the explanation of the underlying mechanism in HFSR. The model of AI-HFSR was showed in **Figure 1**.

### Treatment of AI-HFSR

Due to the lack of clinical trials or investigations, evidence-based approaches of the optimized treatment for HFSR are still missing (52). Therefore, clinical management of targeted therapy-induced HFSR was suggested to take an individualized and multidisciplinary treatment measures (53).

### Patient’s Education and Guidance

Education and guidance for patients with skin side effects in their daily actives is an important aspect for HFSR management that cannot be overlooked. The specific measures include manicure, pedicure, removal of hyperkeratotic skin, and proper handling of eczema and fungal infection before receiving the treatment of apatinib. During the course of therapy, much attention should be paid to the skin care by using alcohol-free moisturizer, avoiding direct contact from hot water (e.g. bath, dish washing), wearing no over-tight garments, minimizing the incidence of exposing to friction (e.g. typing, massage), avoiding excessive physical activities, equipping insole cushions and thick socks and maintaining good personal hygiene.

### Apatinib Reduction and Withdrawal

For HFSR treatment, reduction and withdrawal of apatinib has always been a difficult choice (54). As the CTCAE protocols (30) recommend: For patients with grade I HFSR, continuous administration of apatinib is acceptable with a two weeks’ follow-up visit; For grade II HFSR, treatment with a reduced dosage of apatinib for 7–28 days with timely monitoring is recommended; For grade III HFSR, withdrawal of the medication for at least seven days is recommended, for the purpose of recovering from the toxic reaction to the expected end point of grade I or II.

### Medications

#### Moisturizers

In Shinohara’s report (55), a hydrocolloid dressing containing ceramide with a low-friction external surface was shown to function as a strong skin-protecting cushion through reducing skin friction, buffering pH, and shielding the skin from bacterial. As a result, it effectively relieved the pain caused by HFSR, and prevented the progressing of HFSR towards higher grades.

#### Keratolytic Agents

In China, research by Ai et al. (56) recruited 871 cases, the largest HFSR trial so far, to assess the prophylactic effect of urea cream, combined with the best supportive therapy (BCS), on HFSR. Their results show that such a combination therapy significantly delayed the first onset of HFSR. Similarly, another clinical study that has been carried out in the Guro Hospital of Korea University, is aimed to evaluate the clinical value of urea cream application in treating patients with sorafenib-associated HFSR (57). The outcomes, however, have not been achieved yet.

#### Anti-Proliferation Agents

Previous study has demonstrated that tazarotene can greatly upregulate the expression of TIG-1 (Tazaroten-Induced Gene-1), a trans-membranal protein functioning as a cellular adhesion molecule that may promote cell-to-cell contact, thereby reducing keratinocyte proliferation (58). Moreover, a Japanese doctor, Yamamoto (59), is now leading a clinical study that aims to put forward the application of ascorbyl-2-phosphate magnesium (P-VC-Mg), a cosmetic product, for the treatment of HFSR. Although no results were available yet, his preliminary experimental data (60) have indicated that this highly permeable ascorbic acid derivative could confront against sorafenib-induced hyperproliferation of keratinocytes in a 3D skin model.

#### Traditional Chinese Medicine

Studies by Tian et al. (61) and Zhao et al. (62) demonstrated the efficacy of handling HFSR by successive treatment with the traditional Chinese medications, Danxiong granules and Taohongsiwu. In 2020, Wang et al. (63) reported in a prospective randomized clinical trial study that topical soaking with LC09, a compound granules consisting of Astragali Radix, Angelicae Sinensis Radix, Erodii Herba, Geranii Herba, Arnebiae Radix, and Carthami Flos, could effectively alleviate pain and reduce the severity of HFSR. Moreover, in most cases, the effectiveness of LC09 granules could be achieved within seven days.

#### Other Medications

It has been proved that β-hydroxy-β-methylbutyrate (HMB), L-arginine, and L-glutamine could promote collagen synthesis in the process of wound healing. Recently, Naganuma et al. (64) found that prophylactic administration of an ONS (oral nutritional supplement) containing these ingredients before the initiation of sorafenib treatment could effectively decrease the incidence of HFSR. Other possible medications include analgesics (e.g. lidocaine gel, NSAIDs, GABA agonists) and corticosteroid (e.g. clobetasol).

## Perspective

Apatinib is a newly developed TKI in China. A growing body of studies has validated its clinical efficacy in patients with liver cancer. We believe that, with the forthcoming data of the Phase III study in Chinese patients with liver cancer and a satisfactory cost-effectiveness, extended indications of apatinib approved by the CDFA for targeted therapy of liver cancer can be expected in the near future. Undoubtedly, apatinib will have a great potential in the field of targeting liver cancer in China. Hence, in-depth understanding and optimal clinical management of AI-HFSR, the most common complication of apatinib administration, will always be a challenge for not only hepatologists, but also oncologists, dermatologists, and multidisciplinary teams.

## Author Contributions

All authors provided substantial contribution to the conception, drafting, editing, and final approval of this manuscript. All authors contributed to the article and approved the submitted version.

## Funding

This work were supported by the Chen Xiao-ping Foundation for the Development of Science and Technology of Hubei Provincial (CXPJJH11800001-2018203) and National Natural Science Foundation of China (Grant No.81803157).

## Conflict of Interest

The authors declare that the research was conducted in the absence of any commercial or financial relationships that could be construed as a potential conflict of interest.
